# Isoorientin Promotes Early Porcine Embryonic Development by Alleviating Oxidative Stress and Improving Lipid Metabolism

**DOI:** 10.3390/ani14192806

**Published:** 2024-09-28

**Authors:** Zilong Meng, Jiajun Ren, Chuman Huang, Huimei Huang, Xiuwen Yuan, Yinghua Li, Nam-Hyung Kim, Yongnan Xu

**Affiliations:** Guangdong Provincial Key Laboratory of Large Animal Models for Biomedicine, South China Institute of Large Animal Models for Biomedicine, School of Pharmacy and Food Engineering, Wuyi University, Jiangmen 529000, China; 18119510223@163.com (Z.M.); r2075611233@163.com (J.R.); 18318843832@163.com (C.H.); h2914095135@163.com (H.H.); yeahwwen66@163.com (X.Y.); yhli@wyu.edu.cn (Y.L.); nkkim@wyu.edu.cn (N.-H.K.)

**Keywords:** isoorientin, porcine oocyte, oxidative stress, lipid metabolism

## Abstract

**Simple Summary:**

Isoorientin is an excellent antioxidant and regulator of lipid metabolism. We added this drug to the in vitro medium of porcine embryos and found a significant increase in the developmental rate and quality of the embryos.

**Abstract:**

Isoorientin (ISO) is a natural lignan glycoside flavonoid found in various plants, including Charcot and Stonecrop. ISO exhibits diverse physiological and pharmacological effects, such as antioxidative, anti-inflammatory, hepatoprotective, antiviral, antianxiety, and anti-myocardial ischaemic properties, as well as lipid metabolism regulation. This study investigated the impact of ISO supplementation on oxidative stress and lipid accumulation in porcine early embryos, along with its underlying mechanisms. Porcine embryos were cultured in vitro under different concentrations of ISO (0, 1, 10, and 100 nM). The results revealed that 10 nM ISO significantly enhanced the blastocyst rate and total embryonic cell count in vitro. ISO-treated embryos exhibited reduced reactive oxygen species levels and elevated glutathione levels compared to the untreated group. In addition, ISO treatment significantly increased the expression of the key antioxidant regulator Nrf2, improved mitochondrial function, and reduced lipid droplet accumulation. Concurrently, early embryo autophagy and apoptosis levels decreased. Furthermore, ISO treatment upregulated antioxidant-related genes (*SOD1, SOD2,* and *CAT*) and mitochondrial biogenesis related genes (*NRF1*, *NRF2,* and *SIRT1*), while downregulating lipid synthesis-related genes (*SREBP1* and *FASN*). Additionally, lipid hydrolysis-related genes (*ACADS*) were elevated. These findings collectively suggest that ISO may facilitate early embryonic development in pigs by ameliorating oxidative stress and lipid metabolism.

## 1. Introduction

Reproductive biotechnology, particularly assisted reproductive technology (ART), has advanced rapidly recently, offering significant implications in the field of reproduction. However, the detachment of reproductive biotechnology from the fallopian tubes excludes early maternal–embryo interaction [1], thereby impacting embryo development and resulting in decreased embryo quality [2]. In vitro embryo production efficiency in pigs remains notably low compared to other species [3], primarily due to challenges such as the low maturation rate of porcine oocytes during in vitro maturation (IVM), the high incidence of polyspermia, which is caused by the simultaneous invasion of multiple sperm into a single oocyte, following in vitro fertilisation (IVF), and the suboptimal embryo development and blastocyst quality during in vitro embryo culture, influenced by external factors [4,5]. Given the importance of in vitro-produced pig embryos as research models for human diseases due to their high similarity with humans in terms of body size, physiology, and pathophysiological responses [6], addressing these challenges is imperative.

Embryo in vitro culture (IVC) is a widely employed technique in the in vitro production (IVP) of animal embryos, encompassing the in vitro maturation (IVM) stage [7]. IVC holds promise for application in ART and elucidating biological processes [8,9,10]. However, oxidative stress stemming from reactive oxygen species (ROS) accumulation during IVC significantly hampers embryo development and quality [11], leading to mitochondrial dysfunction [12], lipid metabolism disorders [13], apoptosis [13], and DNA damage [14].

Lipids play a crucial role as an energy source and in biofilm synthesis, necessitating oxidative metabolic homeostasis during embryonic development. In lipid droplets, fatty acids are stored as neutral lipids and released into the cytoplasm as free fatty acids, which undergo oxidative metabolism in the mitochondrial matrix to produce ATP (adenosine triphosphate) [15]. However, high-quality early embryos require oxidative homeostasis of lipids and fatty acids in vivo, with excessive lipid accumulation disrupting this homeostasis and consequently impairing the embryonic developmental process. Excessive lipid accumulation has been associated with high apoptosis rates, lower cold tolerance, and abnormal mitochondrial function. Notably, increased lipid accumulation also stems from embryonic response to various stresses [16].

Discrepancies between in vivo and in vitro environments, particularly in oxygen concentration, along with the need for optimisation of current IVC media, contribute to the inferior quality of in vitro cultured embryos compared to in vivo embryos [17,18]. The addition of antioxidants during IVC shows promise in enhancing in vitro porcine embryo quality and mitigating oxidative stress. For example, melatonin [19,20], chrysin [21], Panax ginseng saponin R1 [22], and isorhamnetin [23] have been demonstrated to improve embryo quality by alleviating oxidative stress. Therefore, identifying drugs to improve oxidative stress and lipid metabolism in early embryos is crucial for the in vitro development of early porcine embryos.

Isoorientin (ISO), a C-glucosyl flavonoid [24], possesses a moderate to strong oxygen-based chemistry and is soluble in water and ethanol [25]. It exhibits anti-inflammatory, antiviral, and antioxidant effects [26]. Additionally, ISO reduces lipid accumulation and scavenges oxygen-free radicals [27]. For instance, ISO attenuated cisplatin-induced nephrotoxicity by inhibiting oxidative stress and apoptosis through SIRT1 (sirtuin 1)/SIRT6 (sirtuin 6)/Nrf-2 (nuclear factor erythroid 2-related factor 2) pathway activation [26]. ISO has also been demonstrated to exert a significant effect on metabolic activity and lipid accumulation in differentiated adipocytes [28]. Notably, ISO ameliorated APAP (paracetamol)-induced hepatotoxicity through the activation of the Nrf2 antioxidant pathway [29]. However, whether ISO improves the quality of early porcine embryos cultured in vitro remains unexplored.

This study aims to investigate the potential of ISO in ameliorating oxidative stress and lipid metabolism during early embryonic development in porcine embryos. We evaluate the blastocyst formation rate, total cell count, apoptosis, and autophagy levels in ISO-exposed early porcine embryos, alongside assessing changes in intracellular oxidative stress, mitochondrial function, lipid metabolism, and related gene expression.

## 2. Materials and Methods

### 2.1. Ethics Statement

The sow ovaries used in this study were obtained from sows that had already been slaughtered in local abattoirs, and there were no ethical issues involved.

### 2.2. Animals and Chemicals

ISO was procured from MedChem Express (#HY-N0767, Monmouth Junction, NJ, USA). Unless specified otherwise, all other reagents were sourced from Sigma-Aldrich (St. Louis, MO, USA). Porcine ovaries were obtained from a local slaughterhouse (Jiangmen, China) for this study. These sows were virgin sows, had never given birth to piglets, and were about 4 to 6 months old. ISO was initially dissolved in dimethyl sulfoxide (DMSO) to achieve a concentration of 20 mM and further diluted with water to a reserve concentration of 500 μM. For experimentation, the reserve concentration was diluted using IVC medium to 1, 10, and 100 nM, ensuring a DMSO concentration of ≤0.005%. The control group did not contain DMSO.

### 2.3. Oocyte Collection and IVM

Porcine ovaries were transported to the laboratory in thermos flasks filled with sterile saline at 37 °C. All porcine ovaries were obtained from sows slaughtered on the same day at the abattoir for non-study purposes. Upon retrieval from the thermos flasks, ovaries were thoroughly washed with sterile saline supplemented with 100 µg/mL penicillin G. Subsequently, the ovaries were rinsed with a 20 mL syringe filled with sterile saline. Follicular fluid containing the cumulus–oocyte complex (COC) was aspirated from follicles measuring 3–8 mm in diameter using a 20 mL sterile syringe and an 18-gauge needle. The extracted follicular fluid was transferred to a shaker tube containing Tyrode lactate 4(2-hydroxyethyl)-1-piperazineethanesulfonic acid (HEPES), and after sedimentation, the supernatant was aspirated, HEPES was added again to clean the COC, and the process was repeated four times. Following cleaning, approximately 100 cumulus more complete COCs per well were placed in a four-well culture plate containing 500ul fresh IVM medium (M199 medium supplemented with 0.022 mg/mL sodium pyruvate, 10% porcine follicular fluid, 0.09 mg/mL L-cysteine, 1% penicillin-streptomycin, 10 IU/mL follicle-stimulating hormone, 20 ng/mL epidermal growth factor, and 10 IU/mL luteinising hormone), and 500 μL of tissue culture oil (ART-4008P, SAGE, Suzhou City, China) was used to cover this IVM medium. Subsequently, the four-well culture plates containing oocytes were incubated at 38.5 °C in an atmosphere of 5% CO_2_ for 44 to 46 h.

### 2.4. Parthenogenetic Activation and In Vitro Embryo Culture

After IVM, COCs were subjected to treatment with 0.2% hyaluronidase and gently repeated blowing 30 times using a pipette gun in a petri dish to remove granulosa cells. Surviving oocytes were selected and placed in an activation solution (300 mM mannitol containing 0.5 mM HEPES, 0.05 mM CaCl_2_-2H_2_O, 0.1 mM MgSO_4_-7H_2_O, and 0.01% polyvinyl alcohol), followed by two rounds of DC pulse stimulation (120 V, 60 us) at intervals of 0.1 s to induce parthenogenetic activation (Cell Fusion Instrument, CFB16-HB, BEX, Tokyo City, Japan). Subsequently, activated oocytes were transferred to an IVC medium (bicarbonate-buffered PZM-5 supplemented with 4 µg/µL bovine serum albumin (BSA) containing 7.5 mg/mL cytarabine B). The oocytes were then incubated at 38.5 °C and 5% CO_2_ for 3–4 h. After 3 h, the oocytes were washed four times with IVC solution that does not contain cytarabine B and distributed into four-well culture plates containing 50 µL small droplets of IVC culture medium (cytarabine B not included), with each well containing 35–50 oocytes; finally, 700 μL of tissue culture oil was used to cover each small droplet. The culture medium in each well was supplemented with different concentrations of ISO (1, 10, and 100 nM), while the control wells received no ISO treatment. Blastocyst formation rates were observed and recorded after seven days, with experiments repeated at least three times.

### 2.5. Measurement of Intracellular ROS and Glutathione (GSH) Levels

Embryos at the four-cell stage, cultured in vitro for 48 h, were selected for assessing ROS and GSH levels. Embryos were incubated in PBS-PVA containing 10 µM 2′,7′-dichlorodihydrofluorescein diacetate (ROS fluorescent probe, H2DCFDA, Beyotime, Shanghai, China) and 10 µM 4-chloromethyl-6,8-difluoro-7-hydroxyc-oumarin (GSH fluorescent probe, CMF2HC, Beyotime, Shanghai, China) at 37 °C for 30 min under light protection. Subsequently, embryos were washed 3–4 times with PBS-PVA, placed in a 4 μL droplet of PBS-PVA, photographed using a fluorescence inverted microscope (Ti2eU; Nikon, Tokyo, Japan), and fluorescence intensity was analysed using ImageJ version 8.0.2 software (NIH, Bethesda, MD, USA).

### 2.6. Mitochondrial Membrane Potential (MMP, ∆Ψ) Assay

Embryos at the four-cell stage, cultured in vitro for 48 h, were placed in PBS-PVA containing 10 µg/mL of 5,5′,6,6′ tetrachloro-1,1′,3,3′-tetraethylbenzimidazolylcarbocyanineiodide (JC-1, Beyotime, Shanghai, China) and incubated for 16 h at 38.5 °C and 5% CO_2_ under light protection. Subsequently, embryos were washed 3–4 times with warm PBS-PVA, placed in 4 μL droplets of PBS-PVA, photographed using a fluorescence inverted microscope (Ti2eU; Nikon, Tokyo, Japan), and the ratio of red to green fluorescence intensities was analysed using ImageJ version 8.0.2 software (NIH, Bethesda, MD, USA). The membrane potential level of the embryos was reflected via the ratio of red fluorescence (j-aggregates) to green fluorescence (j-monomers) of JC-1.

### 2.7. Immunofluorescence Staining

Blastocysts from the IVC medium on day 7 were isolated, washed three to four times with PBS-PVA and fixed in PBS-PVA containing 3.7% paraformaldehyde for 30 min. Subsequently, they were permeabilised in 0.1% Triton X-100 for 30 min, followed by blocking in PBS-PVA containing 3% BSA for 1 h at room temperature. The blastocysts were then incubated with primary antibodies: LC3B (#ab48394, diluted 1:200; Abcam, Cambridge, MA, USA), Nrf2 (#ab31163, diluted 1:200; Abcam, Cambridge, MA, USA), and Caspese-3 (#C8487, diluted 1:100; Sigma-Aldrich, St. Louis, MO, USA). The primary antibodies were incubated overnight at 4 °C away from light. The following day, secondary antibodies were applied. Cells were washed 3–4 times with PBS-PVA and then incubated with goat anti-rabbit antibody (1:500; Abcam; #ab150077; for LC3B and Nrf2 staining) or (1:500; CST; #8889s; for CASPASE3 staining) for one hour at 38.5 °C. Finally, nuclei were labelled with 10 µg/mL Hoechst 33342 for 7 min. After four washes with PBS-PVA, the slices were blocked and photographed using inverted fluorescence microscopy. Fluorescence intensities were analysed using Image J. The relative fluorescence intensity of LC3B was used to assess the level of autophagy, while that of Nrf2 and caspase-3 was used to assess the level of oxidative stress and apoptosis, respectively.

### 2.8. Embryonic Lipid Droplet Staining

Intraembryonic lipid droplets were visualised using a lipid droplet probe. Blastocysts from day 7 were collected, washed four times with PBS-PVA and fixed in 3.7% paraformaldehyde solution for 30 min at room temperature. Subsequently, they were permeabilised in 0.3% TritonX-100 for 30 min at room temperature and then blocked in 3% BSA solution at room temperature for 1 h. After washing four times with PBS-PVA, blastocysts were incubated in a lipid droplet dye solution (BODIPY Lipid Probes Molecular Probes, D3922, Invitrogen, Carlsbad, CA, USA, 10 μg/mL) for 1 h at 37 °C, protected from light. Finally, the nuclei were washed four times with PBS-PVA and stained with 10 μg/mL Hoechst 33342 solution for 7 min to label the nuclei. Following washing with PBS-PVA, the sections were sealed and photographed under an inverted fluorescence microscope. Fluorescence intensity was analysed using ImageJ. The relative fluorescence intensity of the lipid droplets (LDs) was utilised to assess the lipid metabolism levels.

### 2.9. Quantitative Real-Time Reverse Transcription-Polymerase Chain Reaction (qRT-PCR)

Blastocysts from day 7 were collected (approximately 18 blastocysts in both control and treated groups) and rapidly frozen in liquid nitrogen as samples. RNA was extracted and reverse transcribed into cDNA using the Dynamic RNA Direct Purification Kit (Invitrogen) and the RevertAid First Strand cDNA Synthesis Kit (Thermo Fisher Scientific, Waltham, MA, USA), following the manufacturer’s instructions. The KAPA SYBR FAST qPCR Master Mix (2×) Kit (KAPA Biosystems, Sigma-Aldrich, St. Louis, MO, USA) was used to prepare the qRT-PCR system. The reaction mixture comprised 10 μL of KAPA, 0.4 μL of ROX LOW, 0.4 μL of the upstream primer, 0.4 μL of the downstream primer, and 8.8 μL of the cDNA samples, totalling 20 μL. The qRT-PCR conditions included denaturation at 95 °C for 30 s, followed by 40 cycles of denaturation at 95 °C for 5 s, annealing at 60 °C for 15 s, and extension at 72 °C for 30 s. Gene expression was quantified using QuantStudio™ Design & Analysis Software version 1.5.2 and the 2^−ΔΔCt^ method with GAPDH as the reference gene. The primers used are listed in Table 1

### 2.10. Statistical Analysis

Blastocyst rate was calculated as number of blastocysts/number of all embryos in the well. The number of oocytes/blastocysts (N) and the number of independent experimental repetitions (R) are indicated in the corresponding figures. Pairwise statistical significance between the two groups was determined using the *t*-test. Statistical analysis was conducted using SPSS Version 22.0 software (IBM Corporation, Chicago, IL, USA). The results are presented as mean ± standard deviation (SD). Significance levels were denoted as follows: *** *p* < 0.001, ** *p* < 0.01, * *p* < 0.05.

## 3. Results

### 3.1. Effects of Different ISO Concentrations on Porcine Early Embryonic Development

**Figure 1:**
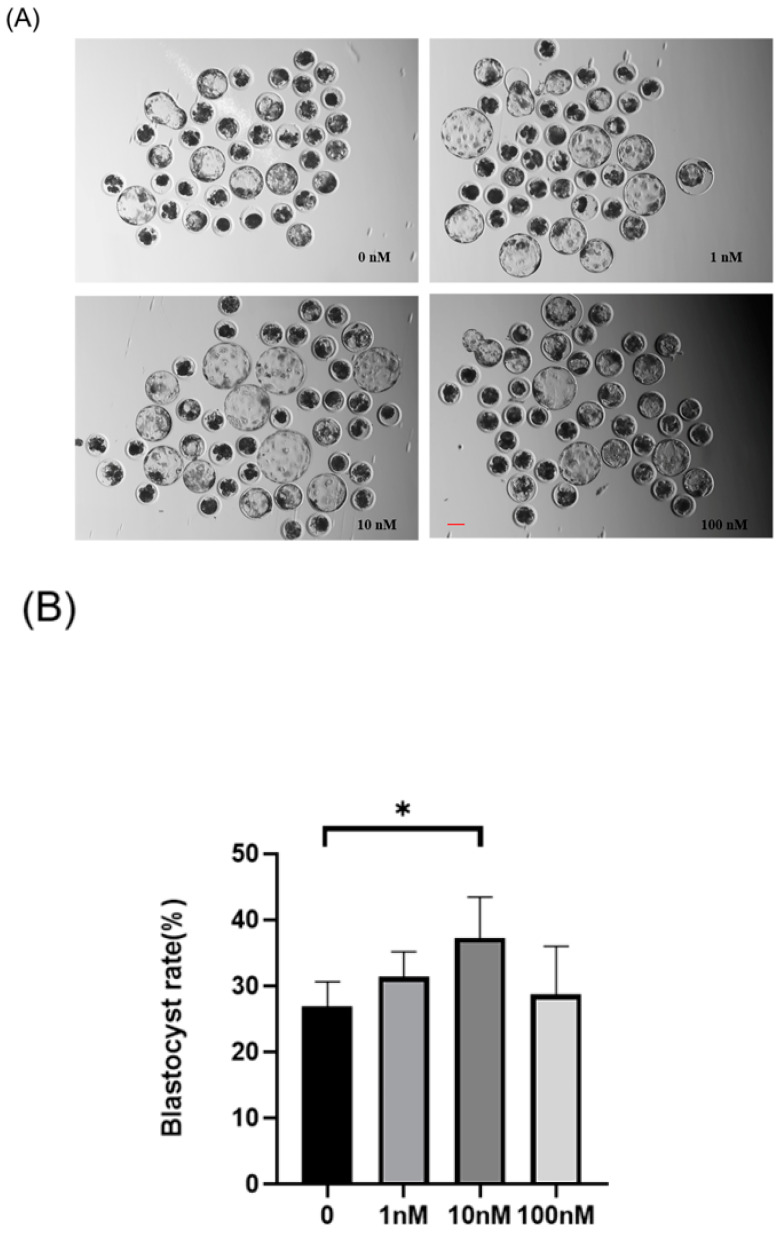

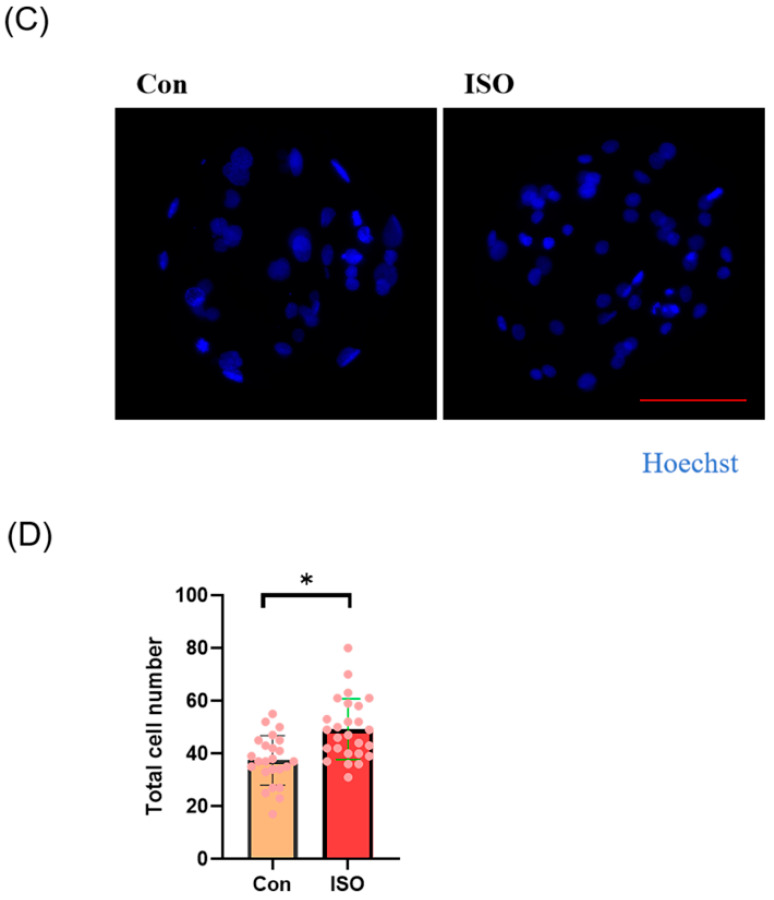
Effects of different concentrations (1, 10, and 100 nM) of isoorientin (ISO) on the formation of porcine blastocysts in vitro. (**A**) Representative images of embryos on day 7 after treatment with 1, 10, and 100 nmol/l of ISO. Scale bar = 100 μm. (**B**) Blastocyst formation rates after treatment with different concentrations of ISO. Day 7 control (*n* = 193) and ISO treatment groups, namely, 1 (n = 195), 10 (n = 199), and 100 (n = 196) nM, R = 5. (**C**) Cell-stained images of blastocysts. Scale bar = 100 μm. (**D**) Blastocysts with (n = 26) or without (n = 24) ISO treatments were stained with Hoechst 33342 and counted (R = 3). The degree of variation was taken as the lowest value of the three replications. * *p* < 0.05.

A preliminary screening of ISO treatment concentrations (1–100 nM) was conducted to determine an optimal concentration range. Multiple replicate experiments were performed with four ISO treatment concentrations: 0, 1, 10, and 100 nM (Figure 1A,B). The blastocyst rates for the 0 (control), 1, 10, and 100 nM groups were 26.92 ± 3.72%, 31.36 ± 3.83%, 37.28 ± 6.14%, and 28.72 ± 7.30%, respectively. A significant increase in the blastocyst rate was observed after ISO treatment at 10 nM (*p* < 0.05), which was consequently chosen as the subsequent experimental treatment concentration.

Additionally, the total cell number of blastocysts on day 7 after ISO treatment was significantly higher in the ISO-treated group compared to the control group (control: 37.42 ± 9.34, ISO: 49.23 ± 11.56, *p* < 0.05, Figure 1C,D).

### 3.2. ISO Enhances Antioxidant Activity in Early Porcine Embryos

**Figure 2:**
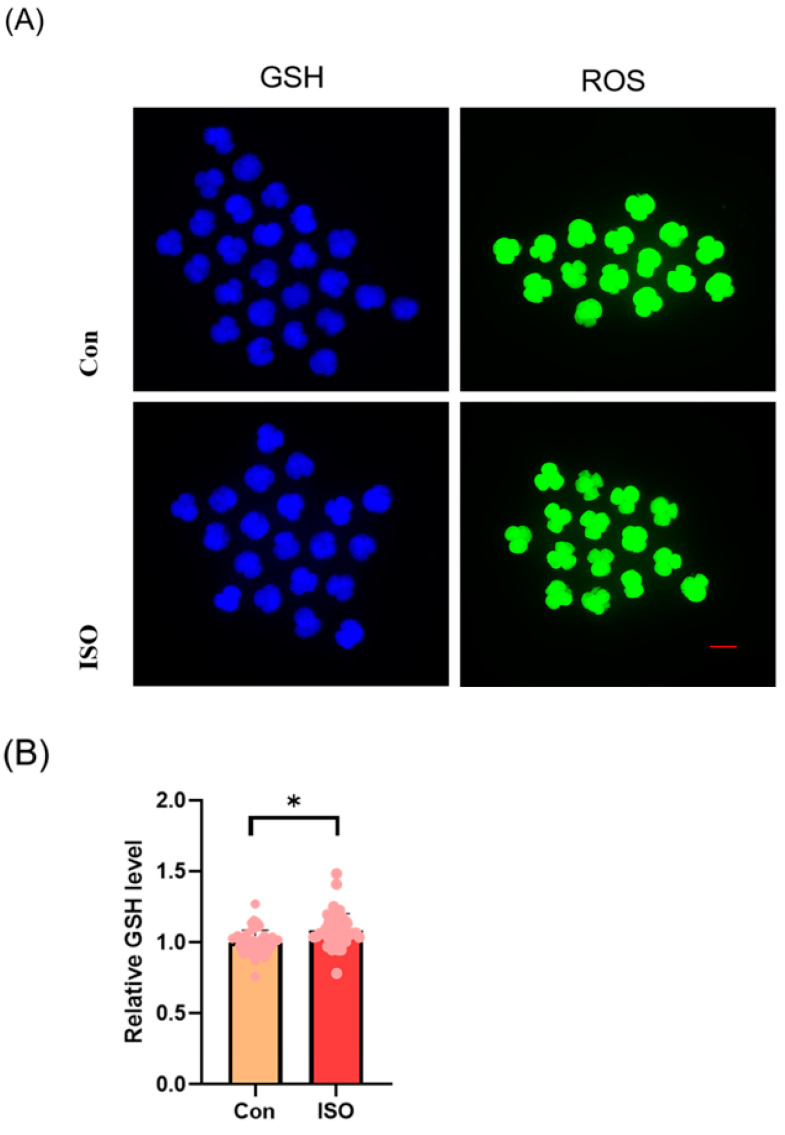

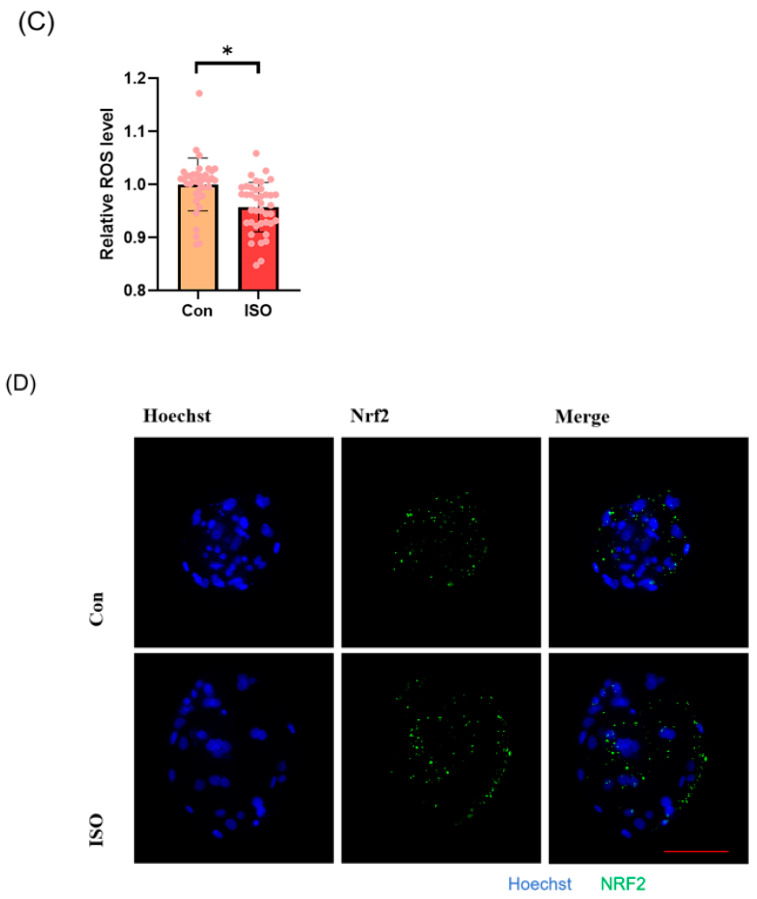

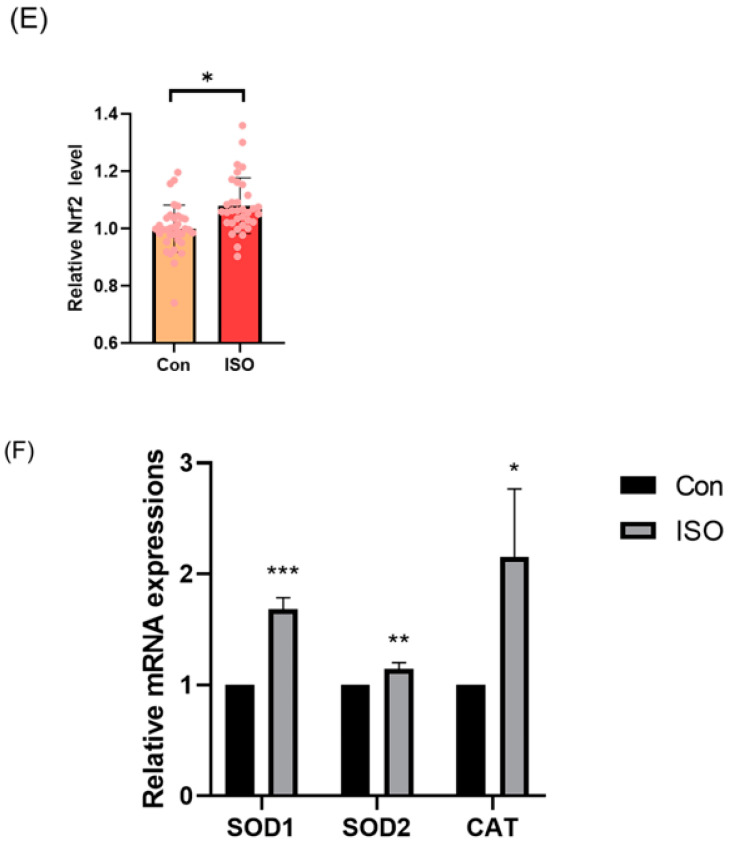
Effect of isoorientin (ISO) on the antioxidant capacity of early embryos. (**A**) Representative micrographs of 4-cell stage embryos stained with CMF2HC (blue) and DCFH (green) for GSH and ROS levels, respectively. Scale bar = 100 mm. (**B**) Relative levels of GSH in embryos with (n = 48) or without (n = 52) ISO treatment (R = 3). (**C**) Relative concentrations of ROS in embryos with (n = 40) or without (n = 38) ISO treatment (R = 3). (**D**) A representative image of blastocysts immunostained for the NRF2 protein. Scale bar = 100 mm. (**E**) Relative levels of NRF2 fluorescence intensity in blastocysts with (n = 34) or without (n = 35) ISO treatment (R = 3). (**F**) Changes in oxidative stress-related gene expression levels after the addition of ISO (R = 3). *** *p* < 0.001, ** *p* < 0.01, * *p* < 0.05.

The levels of GSH and ROS were assessed in embryos at the 4-cell stage, along with the expression of Nrf2, an oxidative stress defence pathway-related factor, at the blastocyst stage. The fluorescence intensity of CMF2HC indicated significantly higher levels of GSH (ISO-treated group: 1.09 ± 0.12, *p* < 0.05, Figure 2A,B), while the fluorescence intensity of DCFH was significantly lower in the ISO-treated group compared to the control group (ISO-treated group: 0.96 ± 0.05, *p* < 0.05, Figure 2A,C), suggesting reduced ROS levels. Moreover, there was a significant increase in Nrf2 fluorescence levels at the blastocyst stage following ISO treatment (ISO-treated group: 1.08 ± 0.10, *p* < 0.05, Figure 2D,E). Consistently, the expression of antioxidant-related genes *SOD1*, *SOD2*, and *CAT* was upregulated in ISO-treated embryos, as revealed via qRT-PCR (1.68 ± 0.11, 1.15 ± 0.06, and 2.15 ± 0.62, *p* < 0.05, *p* < 0.01, *p* < 0.001, Figure 2F).

### 3.3. ISO Improved the Mitochondrial Function in Early-Stage Porcine Embryos

**Figure 3:**
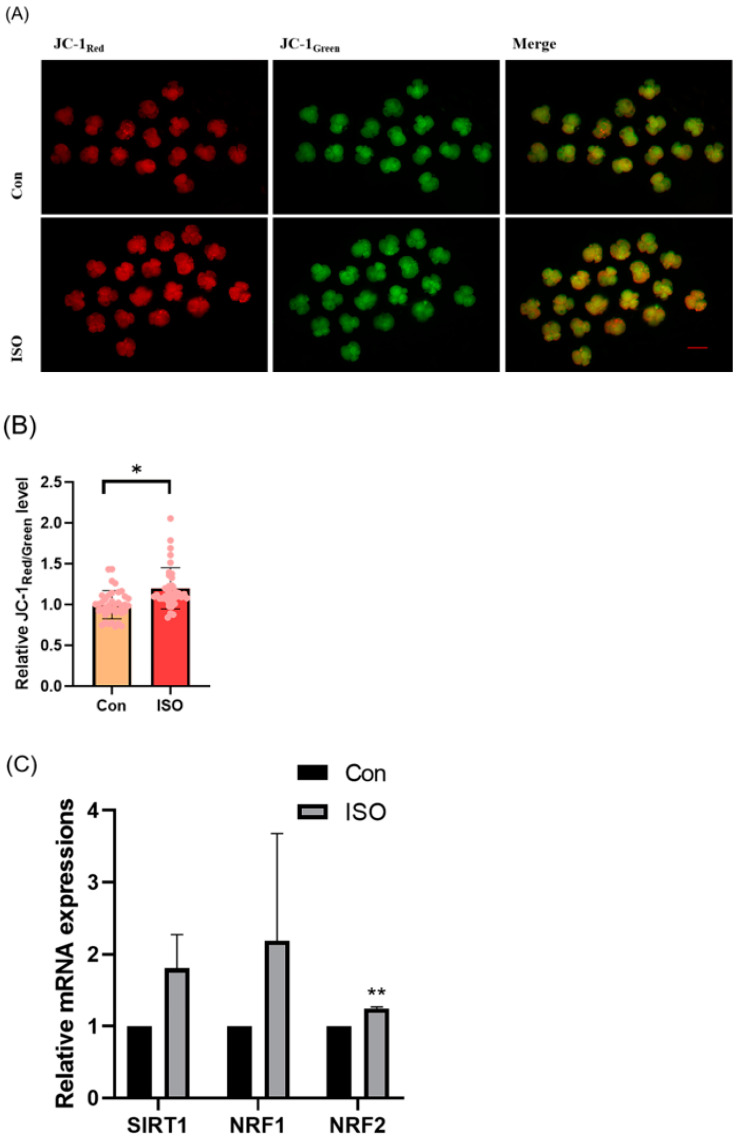
Effect of isoorientin (ISO) on mitochondrial function in early embryos. (**A**) A representative image of 4-cell stage embryos stained with JC-1 to assess mitochondrial membrane potential. Scale bar = 100 mm. (**B**) Relative abundance of mitochondria in embryos with (n = 38) or without (n = 39) ISO treatment (R = 3). (**C**) Changes in mitochondrial gene expression levels following the addition of ISO (R = 3). ** *p* < 0.01, * *p* < 0.05.

The effect of ISO on mitochondrial function in porcine embryos was assessed by measuring the MMP in 4-cell stage embryos collected 48 h after IVC. Relative to controls, the JC-1 red/green fluorescence intensities were 1.20 ± 0.16 times higher in ISO-treated 4-cell stage embryos, indicating a significant increase in MMP (*p* < 0.05, Figure 3A,B). Additionally, mRNA levels of the mitochondrial biogenesis-associated genes *NRF1* and *NRF2*, as well as the mitochondrial synthesis-associated gene *SIRT1*, were significantly higher in ISO-treated embryos compared to the control groups. Specifically, *NRF2* expression was significantly elevated (1.80 ± 0.47, 2.19 ± 1.49, and 1.25 ± 0.02, *p* < 0.01, Figure 3C).

### 3.4. ISO Reduces the Accumulation of LDs in Early Porcine Embryos

**Figure 4:**
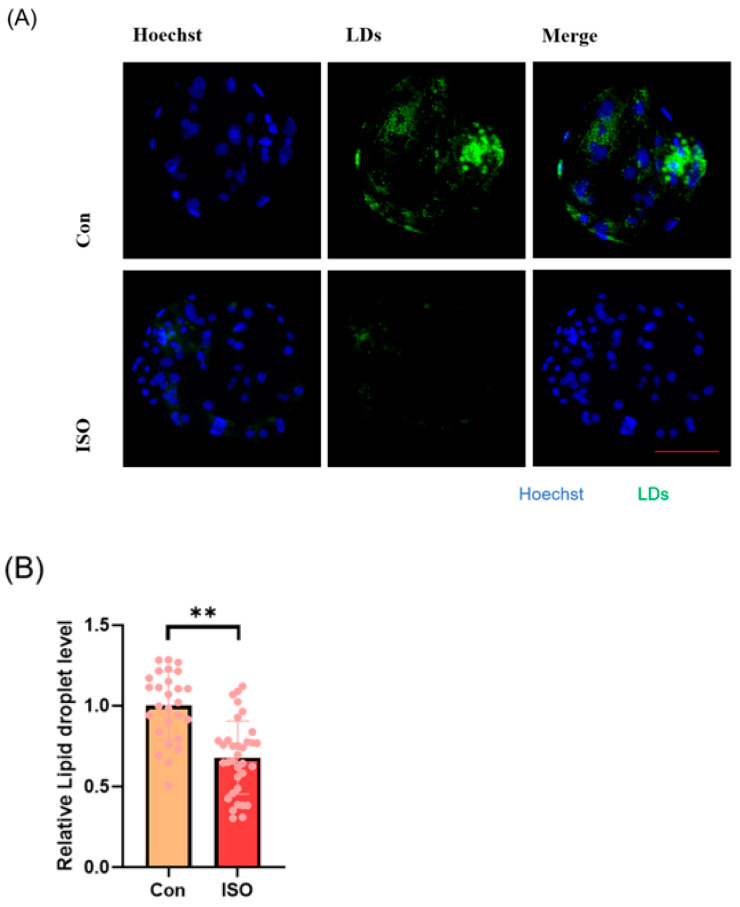

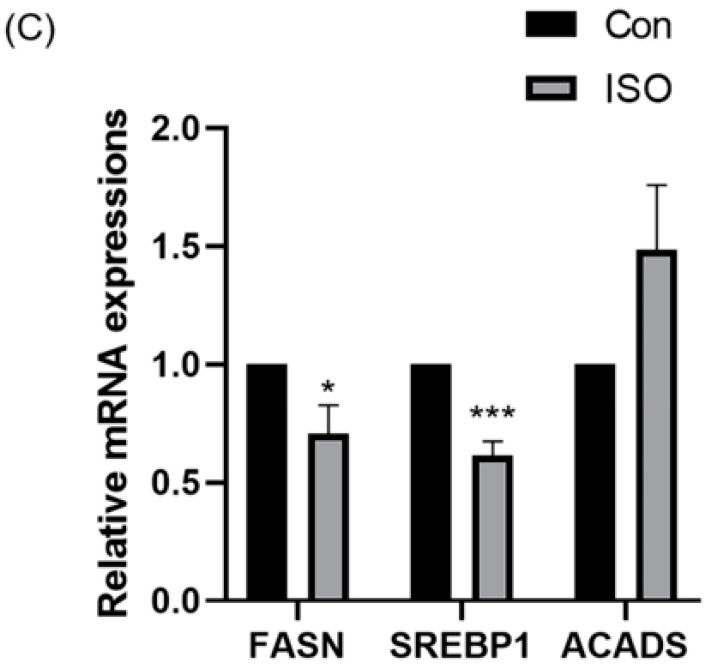
Effect of isoorientin (ISO) on lipid metabolism in early embryos. (**A**) A representative image of lipid droplets (LDs) staining in early embryos. Scale bar = 100 mm. (**B**) Relative level of LD fluorescence intensity in blastocysts with (n = 34) or without (n = 27) ISO treatment (R = 3) (**C**) Changes in lipid metabolism-related gene expression levels following the addition of ISO (R = 3). *** *p* < 0.001, ** *p* < 0.01, * *p* < 0.05.

The effect of ISO on lipid metabolism in embryos was studied via fluorescent labeling of LDs and RT-qPCR analysis of lipid droplet accumulation and mRNA levels of lipid metabolism-related genes in early embryos. ISO treatment resulted in reduced accumulation of LDs in early embryos (0.68 ± 0.23, *p* < 0.01, Figure 4A,B). Furthermore, the expression of lipid metabolism-related genes showed variations, with a significant decrease in the expression of lipid synthesis-related genes *SREBP1* and *FASN*, and a tendency toward increased expression of the lipid hydrolysis-related gene *ACADS* (0.71 ± 0.12, 0.61 ± 0.06, and 1.48 ± 0.28, *p* < 0.05, *p* < 0.001, Figure 4C).

### 3.5. ISO Reduces the Level of Apoptosis in Early Porcine Embryos

**Figure 5:**
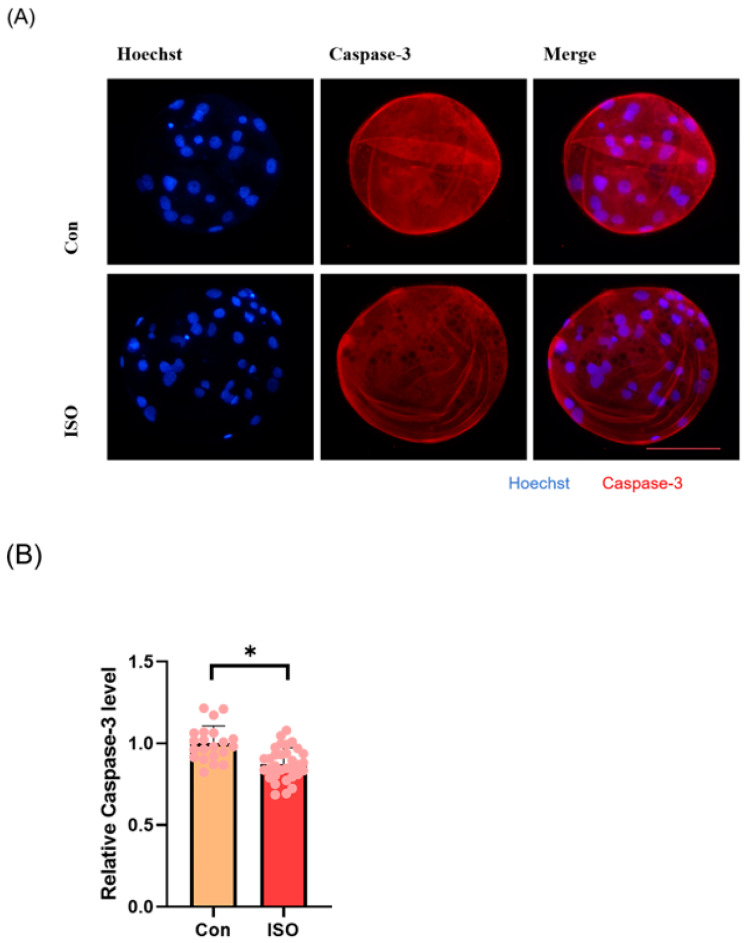

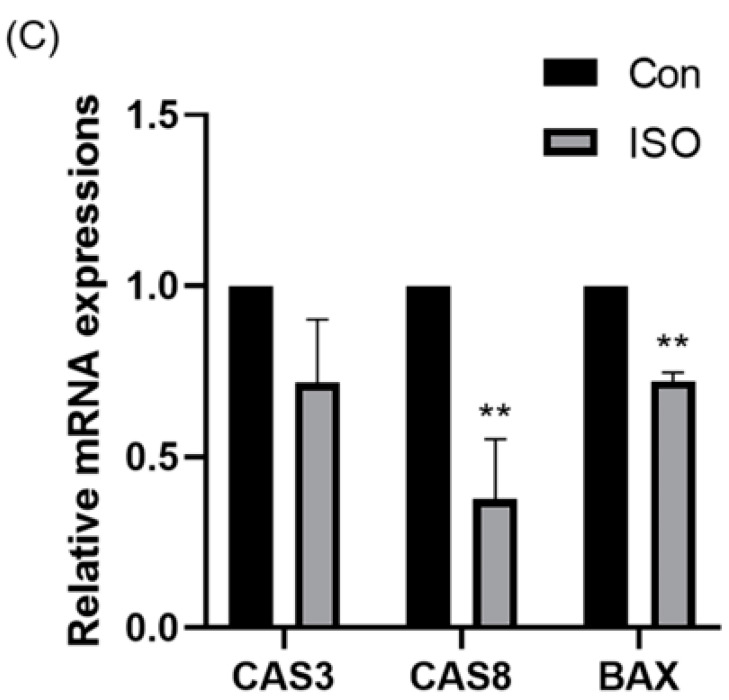
Effect of isoorientin (ISO) on apoptosis in early embryos. (**A**) A representative image of blastocysts immunostained for caspase-3 protein. Scale bar = 100 mm. (**B**) Relative level of caspase-3 fluorescence intensity in blastocysts with (n = 32) or without (n = 21) ISO treatment (R = 3). (**C**) Changes in apoptosis-related gene expression levels following the addition of ISO (R = 3). ** *p* < 0.01, * *p* < 0.05.

To evaluate apoptosis levels in early embryos, blastocyst-stage embryos were subjected to caspase-3 staining. Caspase-3 fluorescence levels were significantly reduced in the ISO-treated group compared to the control group (0.87 ± 0.10, *p* < 0.05, Figure 5A,B). Moreover, qRT-PCR analysis revealed decreased expression levels of pro-apoptosis-related genes *caspase-3*, *caspase-8*, and *BAX* following ISO treatment. Among them, *caspase-8* and *BAX* showed significant reductions, while *caspase-3* exhibited a trend towards decreasing levels (0.72 ± 0.19, 0.38 ± 0.18, and 0.72 ± 0.03, *p* < 0.01, Figure 5C).

### 3.6. ISO Reduces Autophagy Levels in Early Porcine Embryos

**Figure 6:**
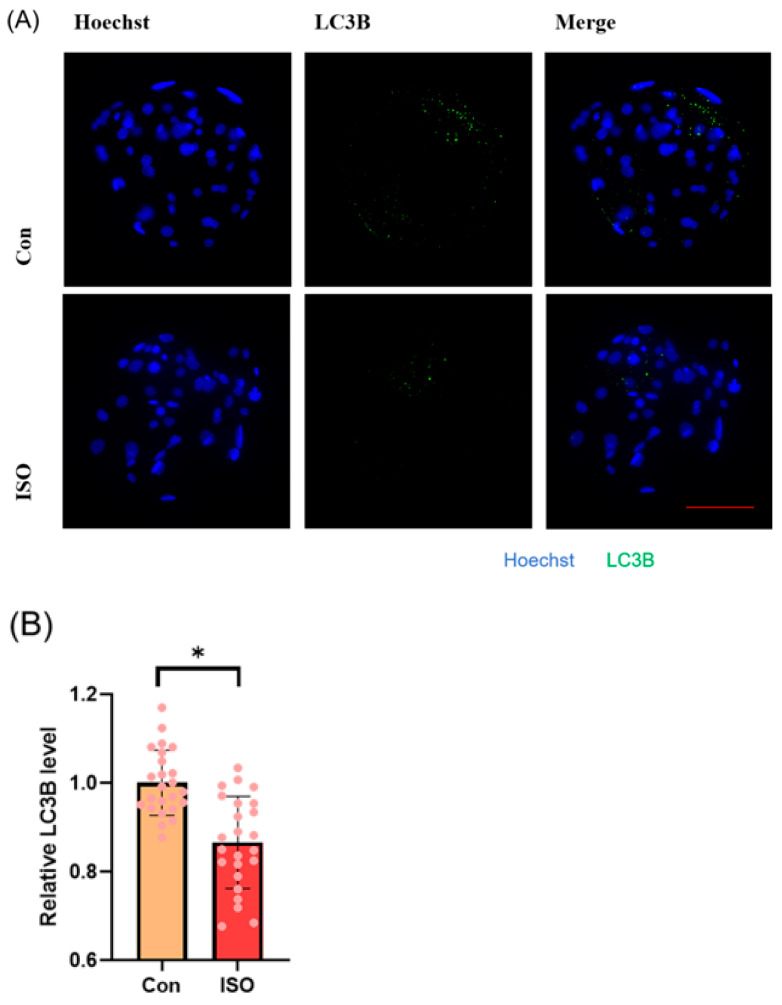

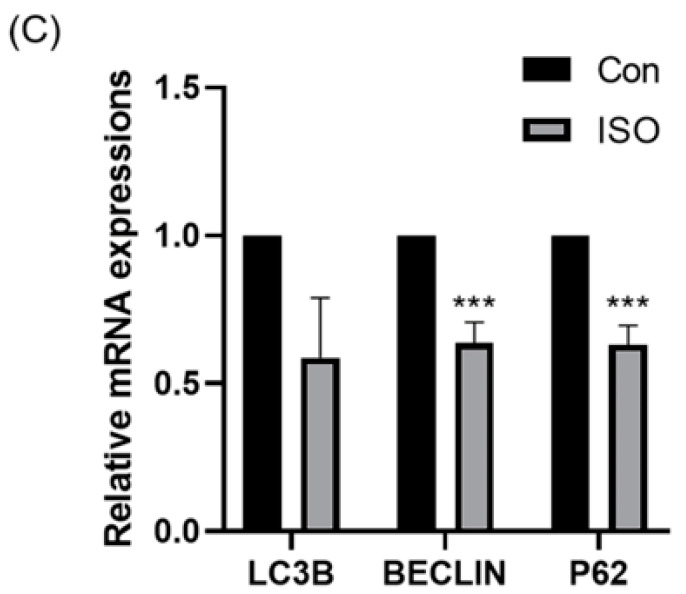
Effect of isoorientin (ISO) on autophagy in early embryos. (**A**) A representative image of blastocysts immunostained for the LC3B protein. Scale bar = 100 mm. (**B**) Relative level of LC3B fluorescence intensity in blastocysts with (n = 24) or without (n = 24) ISO treatment (R = 3). (**C**) Changes in autophagy-related gene expression levels after the addition of ISO (R = 3). *** *p* < 0.001, * *p* < 0.05.

Autophagy level was evaluated by detecting autophagy marker LC3B and analysing the expression of autophagy-related genes. ISO treatment led to a significant reduction in the relative fluorescence intensity level of LC3B (0.87 ± 0.11, *p* < 0.05, Figure 6A,B). Additionally, ISO treatment decreased the expression levels of autophagy-related genes *LC3*, *BECLIN*, and *P62*. Specifically, the expression levels of *BECLIN* and *P62* were significantly reduced (0.59 ± 0.20, 0.64 ± 0.07, and 0.63 ± 0.07, *p* < 0.001, Figure 6C).

## 4. Discussion

In vitro embryo production plays a pivotal role in reproductive technology, offering an efficient selection of superior genetics for transfer or genetic modification compared to in vivo methods [30]. However, embryos produced in vitro are more susceptible to oxidative stress damage due to external environmental stressors such as fluctuating oxygen levels, light exposure, media contaminants, and lack of maternal antioxidant protection [11]. Previous studies have demonstrated that adding antioxidants to porcine embryo cultures can enhance development and reduce ROS levels [31,32]

To enhance the quality of in vitro embryo development, we introduced the flavonoid ISO into the IVC medium. Our study demonstrated that ISO treatment effectively improved the in vitro development of early porcine embryos. This improvement was evidenced by the increased antioxidant capacity, enhanced mitochondrial function, improved lipid metabolism, and reduced levels of apoptosis and autophagy. Overall, these findings suggest a protective role of ISO in in vitro embryo development. Our initial investigation into the blastocyst rate and quality revealed that treatment of 10 nM ISO significantly enhanced blastocyst development, establishing it as the optimal experimental concentration.

Blastocyst formation is a critical milestone in embryo development, characterised by the production of blastocyst fluid and cavity formation. Cell count is an important indicator for evaluating the quality of blastocysts, and blastocysts with higher cell counts are often associated with increased implantation potential [33]. To evaluate the effect of ISO on blastocyst quality, we conducted cell count staining. Our results demonstrated a significantly higher total cell number in ISO-treated blastocysts compared to the control group, indicating the improved quality of early porcine embryos.

Oxidative stress disrupts antioxidant defences, leading to increased ROS levels, which can induce meiotic abnormalities, which are manifested by abnormal spindle morphology, disordered chromosome alignment, mitochondrial dysfunction, and lipid metabolism, ultimately reducing embryo developmental potential [34]. In our study, analysis of ROS and endogenous antioxidant GSH levels in four-cell stage embryos after ISO treatment revealed significantly higher GSH levels and lower ROS levels in the ISO-treated group compared to controls.

The nuclear factor erythroid 2 (NFE2)-related factor 2 (Nrf2) is a member of the cap ‘n’ collar (CNC) subfamily of basic region leucine zipper (bZip) transcription factors. Nrf2 is inhibited under basal conditions by Keap1-controlled ubiquitination–proteasomal degradation and modification of the Keap1, wherein key cysteine thiols of Nrf2 are activated by oxidants and electrophiles. Moreover, activated Nrf2 regulates the expression of various enzymes and signalling proteins, consequently regulating drug metabolism, antioxidant defence mechanisms, and oxidative signalling [35].

In this study, blastocyst-stage embryos were analysed for Nrf2 fluorescence levels, revealing a significant increase in Nrf2 fluorescence in the ISO-treated group compared to the control group. This finding suggests that ISO may ameliorate oxidative stress through the Nrf2 pathway. Additionally, ISO treatment effectively increased the relative mRNA levels of antioxidant-related genes *SOD1*, *SOD2*, and *CAT*. These results indicate that ISO treatment can enhance the antioxidant capacity of early porcine embryos.

Mitochondrial function is closely intertwined with oxidative stress, and mitochondrial dysfunction often precedes oxidative damage, impairing oocyte quality and early embryonic development [36,37]. In this study, four-cell stage embryos underwent JC-1 staining to assess mitochondrial function by analysing the red/green ratio. We observed a significantly higher red/green ratio in the ISO-treated group compared to the control group. Concurrently, ISO treatment upregulated the mRNA levels of mitochondrial biogenesis-related genes *NRF1* and *NRF2*, as well as the mitochondrial synthesis gene *SIRT1*. NRF2, distinct from Nrf2, refers to the nuclear respiratory factor NRF-2 and plays a pivotal role in coordinating mitochondrial biogenesis with nuclear function [38]. These results collectively suggest that ISO treatment enhances mitochondrial function in early porcine embryos.

Compared to other domestic animals, porcine oocytes and embryos exhibit a high lipid content, which is stored as droplets in the cytoplasm [15,39,40,41,42]. These LDs, mainly composed of neutral lipids such as triglycerides (TG) and cholesteryl esters (CE), are crucial for lipid metabolism, serving as substrates for energy production, membrane components, and signalling lipids [43,44]. During early porcine embryo development, the LD content gradually decreases, potentially due to enhanced β-oxidation supporting the increased energy demand associated with blastocyst formation, blastocyst lumen expansion, and hatching [45]. Our study evaluated LD levels in blastocyst-stage embryos, revealing a significant reduction in LDs in the ISO-treated group compared to the control group. Furthermore, ISO treatment downregulated the mRNA expression of adipogenesis-related genes *SREBP1* and *FASN*, while upregulating the mRNA level of the lipid hydrolysis-related gene *ACADS*. These findings indicate that ISO treatment improved lipid metabolism in early porcine embryos.

It is widely acknowledged that oxidative stress induces apoptosis. However, lipid metabolism is inextricably linked to apoptosis. Certain lipids directly activate cysteine asparaginase (caspase), triggering programmed cell death. For example, triglycerides [46], lysophosphatidylcholine [47], lipopolysaccharides [48], and cholesterol [49] can induce caspase-1 activation. Apoptosis induced by fatty acids and their derivatives involves significant activation of caspase-2, -3, -6, -7, -8, and -9 [50,51]. Hence, studying apoptosis levels in early porcine embryos post ISO treatment becomes essential. In our study, we evaluated apoptosis levels by analysing caspase-3 fluorescence levels, revealing significantly lower fluorescence levels post ISO treatment compared to the control group. Furthermore, ISO treatment reduced the mRNA levels of pro-apoptotic genes *CASPASE3*, *CASPASE8*, and *BAX*.

Autophagy, a cellular self-protection mechanism, involves lysosomal degradation of aged, damaged, or denatured macromolecules or organelles due to external stimuli, thus aiding cellular metabolism. Cellular autophagy levels are closely related to lipid metabolism and oxidative stress levels [52,53]. Lower levels of autophagy indicate an improved culture environment. Therefore, investigating autophagy levels in ISO-treated porcine early embryos becomes crucial. We explored the level of LC3B, a cellular autophagy marker, and observed significantly lower relative fluorescence intensity post ISO treatment compared to the control group. Additionally, mRNA levels of autophagy-related genes, *LC3B*, *BECLIN1*, and *P62*, decreased after ISO treatment. These findings suggest that ISO treatment improved the IVC medium environment and alleviated cellular autophagy levels.

## 5. Conclusions

**Figure 7:**
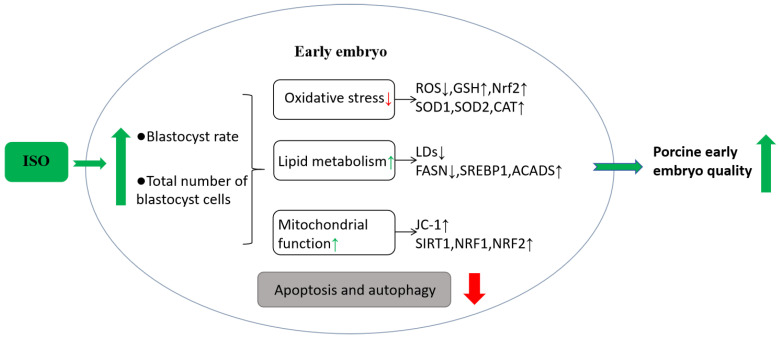
Hypothetical model of isoorientin (ISO) in early porcine embryos. Effects of ISO treatment on early embryos. ISO exposure to early embryos improves oxidative stress and lipid metabolism, enhances mitochondrial function, and positively affects embryonic developmental potential, ultimately decreasing cellular autophagy and apoptosis levels.

The present study demonstrates that ISO possesses the ability to alleviate oxidative stress and improve lipid metabolism, improve mitochondrial function, and reduce apoptosis and autophagy levels in early porcine embryos, thereby promoting their early development (Figure 7). Consequently, we speculate that ISO can optimise embryonic IVC media systems, laying the groundwork for enhanced in vitro embryo production.

## Figures and Tables

**Table 1:** Sequences of primers used in RT-qPCR.

Genes	Sequences 5′–3′	Base
GAPDH	F:TTCCACGGCACAGTCAAG	18
	R:ATACTCAGCACCAGCATCG	19
SOD1	F:CAAAGGATCAAGAGAGGCACG	21
	R:CGAGAGGGCGATCACAGAAT	20
SOD2	F:TTCTGGACAAATCTGAGCCCTAACG	25
	R:CGACGGATACAGCGGTCAACTTC	23
CAT	F:AACTGTCCCTTCCGTGCTA	19
	R:CCTGGGTGACATTATCTTCG	20
LC3B	F:TTCAAACAGCGCCGAACCTT	20
	R:TTTGGTAGGATGCTGCTCTCG	21
BECLIN	F:AGGAGCTGCCGTTGTACTGTTCT	23
	R:TGCTGCACACAGTCCAGGAA	20
P62	F:AAGAACGTAGGGGAGAGTGTG	21
	R:TTCCCTCCATGTTCCACGTC	20
CASPASE3	F:AGAATTGGACTGTGGGATTGAGACG	25
	R:GCCAGGAATAGTAACCAGGTGCTG	24
CASPASE8	F:GCCTCGGGGATACTGTTTGA	20
	R:CGCTGCATCCAAGTCTGTTC	20
BAX	F:GGACTTCCTTCGAGATCGGC	20
	R:GCGTCCCAAAGTAGGAGAGG	20
SIRT1	F:GAGAAGGAAACAATGGGCCG	20
	R:ACCAAACAGAAGGTTATCTCGGT	23
NRF1	F:CCTGTGAGCATGTACCAGACT	21
	R:ACTGTTCCAACGTCACCACCT	21
NRF2	F:AGCGGATTGCTCGTAGACAG	20
	R:TTCAGTCGCTTCACGTCGG	19
FASN	F:GGTTCCAAGGAGCAAGGTGT	20
	R:ATGTACTCCAGGGACTCGGG	20
SREBP1	F:CACGGAGGCGAAGCTGAATA	20
	R:TCTGGTTGCTCTGCTGAAGG	20
ACADS	F:TCATCAAGGAGCCGGCAATG	20
	R:CAAGATCYGGATGGCCTGGTG	21

F = forward primer; R = reverse primer; RT-qPCR = reverse transcription followed by quantitative polymerase chain reaction.

## Data Availability

All results are displayed in charts. For more information, please contact the corresponding author.
